# Objectively evaluated physical activity and sedentary time in primary school children by gender, grade and types of physical education lessons

**DOI:** 10.1186/s12889-018-5910-y

**Published:** 2018-08-02

**Authors:** Chiaki Tanaka, Maki Tanaka, Shigeho Tanaka

**Affiliations:** 1 Division of Integrated Sciences, J. F. Oberlin University, 3758 Tokiwamachi, Machida, Tokyo, 194-0294 Japan; 2 Department of Child Education, Kyoto Seibo College, 1 Taya-cho, Fukakusa Fushimi-ku, Kyoto, 612-0878 Japan; 3 Department of Nutrition and Metabolism, National Institute of Health and Nutrition, National Institutes of Biomedical Innovation, Health and Nutrition, 1-23-1 Toyama, Shinjuku-ku, Tokyo, 162-8636 Japan

**Keywords:** Physical activity, Sedentary time, Exercise, Sports, Sitting

## Abstract

**Background:**

During the typical school day, physical education (PE) gives children an opportunity for physical activity (PA) and reduces their sedentary time, but little is known about objectively evaluated PA and sedentary time during PE lessons and the differences across genders and grades. There is also a lack of research comparing PA and sedentary time among the different types of PE lessons. The primary aim of this study was to examine primary school students’ moderate-to-vigorous PA (MVPA) levels and sedentary time during PE and whether there are gender or grade differences in a cross-sectional study. The secondary aim was to determine which types of PE lessons are best for increasing PA and reducing sedentary time.

**Methods:**

Objectively evaluated MVPA and sedentary time during PE lessons in Japan with a triaxial accelerometer (Active style Pro HJA-350IT, Omron Healthcare) in girls (*n* = 221) and boys (*n* = 181). Minutes of sedentary time were categorized into metabolic equivalents (METs): categories ranged from sedentary time (METs ≤1.5) to MVPA (METs ≥3.0). Time tables and PE lesson types were evaluated using logs maintained by the class teachers.

**Results:**

Time spent in MVPA and sedentary time were 27.3 and 24.3%, respectively. After adjustments for grade, relative body weight and school, boys spent significantly more time in MVPA compared with girls, but with an estimated mean difference of approximately 1 min. After adjustment for gender, relative body weight and school, the younger grades (1st and 2nd) spent significantly more time in MVPA and significantly less time in sedentary time compared with other grades. Moreover, after adjustments for gender, grade, relative body weight and school, the time spent in MVPA during gymnastic and track and field lessons was significantly lower than that during ball game lessons. Sedentary time during gymnastic lessons was significantly longer than in track and field and ball game lessons.

**Conclusions:**

Children did not engage in much MVPA and also spent time in sedentary time during PE, but there are no gender differences. The children were most active during ball game lessons. Therefore, it is important to increase MVPA and reduce sedentary time during PE in both genders.

## Background

The prevalence of participation in organized sport in Japanese primary school children was reported as approximately 40% in girls and approximately 60% in boys by the Report of Survey on Physical Strength and Athletic Performance of Japan Sports Agency [1]. However, during the school day, physical education (PE) should provide an opportunity for children to perform physical activity (PA), and PE also provides an opportunity for children to reduce sedentary time during the school day. However, a recent systematic review reported that little is known about objectively evaluated PA during PE and differences across genders and grades [2].

The Ministry of Education, Culture, Sports, Science and Technology (MEXT) sets educational curriculum guidelines for all Japanese elementary school children including the content of PE lessons and the number of classes, as well as guidelines for school infrastructure and equipment [3]. According to the national educational curriculum guideline, the stated aim of PE is to improve physical fitness and to promote a positive attitude toward exercise as a lifelong PA. However, it is worth noting that a recent global systematic review of the moderate-to-vigorous PA (MVPA) content of PE classes suggested that only a minority of time in PE classes was spent in MVPA [2]. When measured using accelerometers, children were shown to spend 32.6% (95% confidence interval [CI]: 5.9–59.3) of their PE lesson time in MVPA. In addition, the systematic review showed that only one study reported a gender difference during PE lessons [2]. Moreover, very few studies have compared MVPA between the types of exercises performed during PE with using accelerometry in primary school children [2]. Wood and Hall [4] found that children aged 8–9 years engaged in significantly higher MVPA during team games (e.g., football) compared to movement activities in PE lessons (e.g., dance). However, they did not report on the levels of sedentary time during PE lessons. Telford et al. [5] reported the duration of PA (sitting, standing, walking and very active times) during PE lessons in randomly selected children in primary schools. However, PA was evaluated by observers, and subjective evaluation of PA has limited accuracy. On the other hand, Nettlefold et al. reported that the amount and percentage of MVPA, LPA and SB (girls: 13.0, 5.6 and 73.0%, boys: 11.4, 5.6 and 74.5%, respectively) as evaluated with the ActiGraph GT1M during PE lessons in 8–11-year-old Canadian boys and girls [6].

Previous studies proposed using prediction models of METs (metabolic equivalents) for children that included data from accelerometers. In such models, the slope and intercept of ambulatory activities, such as walking and running, differ from those of non-ambulatory activities, such as ball tossing, aerobic dance, and playing with blocks [7–12]. Based on the variability across accelerometer counts, Hikihara et al. [7] showed discrimination between ambulatory activities, such as continuous walking or jogging, and non-ambulatory activities, which include lifestyle activities in primary school children. Considering the complexity of PA of children during PE lessons, it is necessary to take into account whether PA is ambulatory or non-ambulatory. The primary aim of this study was to measure light PA (LPA) to vigorous PA (VPA) levels and sedentary time during PE in primary school students and assess whether there are gender or grade differences in a cross-sectional study. The secondary aim was to determine which types of PE lessons best promote MVPA and reduce sedentary time.

## Methods

Our study sample included 569 Japanese primary school children from 14 public primary schools in the urban areas of Tokyo, Kanagawa and Kyoto. The children were invited to participate in the study at their school by the distribution of leaflets. All participants and their parents gave written informed consent to participate in this study and the Ethical Committee of J. F. Oberlin University approved the study protocol (No.12023). Anthropometric measurements, sedentary time, and PA data were collected from June 2012 to January 2015 during each school year. The initial sample included 569 participants, but 52 children were subsequently withdrawn for the following reasons: revocation of the agreement (*n* = 8); history of conditions that can affect PA, such as respiratory disease or heart disease (*n* = 28); and lacking questionnaire data (*n* = 16). Data analyses were performed on the remaining 517 children. There was no significant difference in the relative weights of the study group and the children who withdrew from the study. The mean age of the study group was 9.2 (SD 1.5) years.

### Objective measurement of sedentary time and physical activity

We measured the children’s PA and sedentary time during PE lessons with a triaxial accelerometer (Active style Pro HJA-350IT, Omron Healthcare, Kyoto; dimensions 74 × 46 × 34 mm and weight 60 g including batteries). This device is described in detail elsewhere [7]. The participants wore their accelerometers on the left side of their waists as instructed by the research assistant and were monitored continuously for 1 week at school. The participants were requested to wear the devices at all times, except under special circumstances, such as dressing and bathing, and data from participants with PE class data and 600 min/d or more for at least 2 days were extracted. Nonwear time was defined as consecutive zero count of 60 min or more. The epoch length, nonwear time definition, and valid days criteria applied in the present study are within the recommended range or the range commonly used [13]. We found PE lesson time for the measurement period in every participant with using timetables by class teachers. We included PE lessons in which 45 min of wearing time had accrued. Valid number of class was ≥ one lesson in analysis. We calculated the synthetic acceleration of all three axes with the signals obtained preceding and following high-pass filtering. We then calculated the ratio of unfiltered to filtered acceleration to identify non-ambulatory activities (e.g., tossing a ball, and cleaning and clearing away) and ambulatory activities (e.g., walking and running). We predicted the MET values from the filtered synthetic acceleration at each 10 s period. Because the predictive equations for the Active style Pro had only been established in an adult population, MET values recorded by this accelerometer are overestimated in primary school children [7]. To compensate, we used the conversion equations below for primary school children, based on a previous study [7]:

$$ {\displaystyle \begin{array}{c}\mathrm{Ambulatory}\ \mathrm{activities}:0.6237\times \mathrm{MET}\ \mathrm{value}\ \mathrm{of}\ \mathrm{Active}\ \mathrm{style}\ \mathrm{Pro}+0.2411\\ {}\mathrm{Non}-\mathrm{ambulatory}\ \mathrm{activities}:0.6145\times \mathrm{MET}\ \mathrm{value}\ \mathrm{of}\ \mathrm{Active}\ \mathrm{style}\ \mathrm{Pro}+0.5573\end{array}} $$

### Time tables and types of PE lessons

Class teachers of study participants were asked to log time tables and types of PE lessons during measurement periods in a log sheet. Moreover, numbers of students their classes were asked.

### Anthropometric measurements

The participants’ body height and weight were measured without shoes but with clothing to the nearest 0.1 cm and 0.1 kg, respectively. Children wore shorts or skirts and t-shirts and shirts. We measured some children’s clothes, and the average weight was 0.7 kg. The weight of clothes (0.7 kg) was subtracted from the measured body weight with clothes and the subtracted weight was used as net body weight. Body mass index was calculated as weight in kilograms divided by height in meters squared. Weight status was categorized into normal weight, overweight/obese, or thin per the Japanese weight cut-offs based on the national reference data for Japanese children [14].

The relative weight was calculated as [body weight (kg) - standard weight for gender, age, and height (kg)] / standard weight (kg)×100 (%)

$$ {}^{\ast}\mathrm{standard}\ \mathrm{weight}\left(\mathrm{kg}\right)=\mathrm{a}\times \mathrm{measured}\ \mathrm{body}\ \mathrm{height}\left(\mathrm{cm}\right)-\mathrm{b} $$

a and b are gender- and age-specific.

The cut-off values for weight categories were defined as follows: overweight/obesity combined, ≥ + 20%; normal weight, − 20 to + 20%; thin, ≤ − 20%.

### Analyses

The time spent being sedentary and in each PA intensity each day was calculated using METs for each participant: average minutes spent in sedentary time (METs ≤1.5), LPA (METs 1.6–2.9), MPA (METs 3.0–5.9), MVPA (METs ≥3.0) and VPA (METs ≥6.0). For the primary aim of the study, the mean values were then calculated for each participant attending PE lessons twice or more per week. PA assessed by the accelerometer was presented as PA status for ambulatory activity or non-ambulatory activity in intensity-specific categories (LPA, MPA, MVPA, and VPA).

One hundred fifteen children were withdrawn from the study for the following reasons: removing the accelerometer during the PE lesson (number of PE lessons = 55); nonattendance of PE lessons (number of PE lessons = 34); 40 min of PE lesson time (number of PE lessons = 10), because a PE lesson time is 45 min/lesson in Japanese primary schools [15]; and no PE lesson during measurement periods (number of PE lessons = 58). Data analysis was carried out on the remaining 402 children. The total number of PE lessons was 714 (1–2 lessons/participant).

For the first study aim, the associations between PAs or sedentary time and gender (girls and boys) were assessed by analysis of covariance (ANCOVA) adjusted for grade, relative body weight and school. The associations between PAs or sedentary time and grades (lower grade: 1st and 2nd grades, middle grade: 3rd and 4th grades, higher grade: 5th and 6th grades) were analyzed by ANCOVA adjusted for gender, relative body weight and school.

For the second study aim, data for girls and boys were analyzed together, because there was no interaction between gender and grade or relative body weight. Each PE lesson was classified into one of the 5 types of lessons defined by the education guidelines for PE for Japanese primary school children by the Ministry of Education, Culture, Sports, Science and Technology [3]: 1) Physical Fitness, 2) Apparatus Gymnastics (1st and 2nd grades: a type of physical play in which an instrument is used, 3rd to 6th grades: gymnastics), 3) Track and field (1st and 2nd grades: physical play with run and jump, 3rd and 4nd grades: exercise with run and jump, 5th and 6th grades: track and field), 4) ball games (1st to 4nd grades: games, 5th and 6th grade: ball games), and 5) Expressive activity (1st and 2nd grades: expressive rhythm play, 3rd to 6th grades: expressive activity).The children were tertiled based on the class size. Firstly, the associations between PAs or sedentary time and the type of PE lessons were analyzed. Secondly, the associations between PAs or sedentary time and the number of students in each lesson were analyzed. Finally, the associations between PAs or sedentary time every type of lesson and class size were also analyzed. In all three analyses, ANCOVA adjusted for gender, grade, relative body weight, and school was used.

Results are shown as means ± standard deviation, unless otherwise noted. Statistical analysis was performed with IBM SPSS Statistics 23.0 for Windows (IBM Co., Tokyo, Japan). All statistical tests were regarded as significant when *p* values were ≤0.05.

## Results

### Characteristics of the study participants

Table 1 shows the characteristics of the study participants and time spent at different intensity levels and total time for ambulatory and non-ambulatory activity and sedentary time during PE lessons. Five percent of the participants (girls 2.3%, boys 7.8%) were overweight/obese.

**Table 1:** Physical characteristics of the participants and time in physical activity and sedentary behavior during physical education lessons

		All		Girls		Boys	
		Mean ± SD (n: 402)	95% CI	Mean ± SD (n: 221)	95% CI	Mean ± SD (n: 181)	95% CI
Age (years)		9.2 ± 1.5	9.1–9.3	9.2 ± 1.5	9.0–9.4	9.1 ± 1.5	8.9–9.4
Height (cm)		131.5 ± 10.0	130.5–132.5	131.7 ± 10.7	130.3–133.1	131.2 ± 9.0	129.9–132.5
Weight (kg)		28.5 ± 6.8	27.8–29.1	28.1 ± 6.6	27.2–29.0	28.9 ± 6.9	27.9–29.9
Body Mass Index (kg/m^2^)		16.3 ± 2.2	16.0–16.5	16.0 ± 1.9	15.7–16.2	16.6 ± 2.4	16.2–17.0
Relative body weight (%)		−2.8 ± 11.9	−4.0--1.6	−4.1 ± 10.7	−5.5--2.7	−1.3 ± 13.1	−3.2-0.7
Weight status (overweight and obese:%)		19 (4.7%)		5 (2.3%)		14 (7.8%)	
Weight status (thin:%)		12 (3.0%)		9 (4.1%)		3 (1.7%)	
PE (min/lesson)		(PE lessons: 714)		(PE lessons: 388)		(PE lessons: 326)	
LPA	Non-ambulatory (min)	11.3 ± 4.9	10.9–11.7	11.7 ± 4.8	11.2–12.2	10.9 ± 4.9	10.3–11.4
	Non-ambulatory (%)	25.1 ± 10.9	24.3–25.9	26.0 ± 10.8	24.9–27.0	24.1 ± 11.0	22.9–25.3
	Ambulatory (min)	10.5 ± 4.5	10.2–10.8	10.2 ± 4.4	9.7–10.6	10.9 ± 4.7	10.4–11.4
	Ambulatory (%)	23.3 ± 10.1	22.6–24.1	22.6 ± 9.8	21.6–23.5	24.2 ± 10.4	23.1–25.4
	Total (min)	21.8 ± 5.3	21.4–22.2	21.8 ± 5.5	21.3–22.4	21.8 ± 5.1	21.2–22.3
	Total (%)	48.4 ± 11.8	47.6–49.3	48.5 ± 12.2	47.3–49.8	48.3 ± 11.3	47.1–49.6
MPA	Non-ambulatory (min)	2.5 ± 2.1	2.4–2.7	2.5 ± 2.1	2.3–2.7	2.5 ± 2.0	2.2–2.7
	Non-ambulatory (%)	5.6 ± 4.6	5.2–5.9	5.6 ± 4.6	5.2–6.1	5.5 ± 4.5	5.0–6.0
	Ambulatory (min)	7.2 ± 4.6	6.9–7.5	6.9 ± 4.3	6.5–7.4	7.5 ± 4.8	7.0–8.0
	Ambulatory (%)	16.0 ± 10.1	15.2–16.7	15.4 ± 9.6	14.5–16.4	16.6 ± 10.6	15.5–17.8
	Total (min)	9.7 ± 5.0	9.3–10.1	9.5 ± 4.8	9.0–10.0	9.9 ± 5.2	9.4–10.5
	Total (%)	21.5 ± 11.1	20.7–22.4	21.1 ± 10.7	20.0–22.1	22.1 ± 11.6	20.8–23.4
VPA	Non-ambulatory (min)	0.7 ± 1.1	0.7–0.8	0.6 ± 0.9	0.6–0.7	0.8 ± 1.3	0.7–1.0
	Non-ambulatory (%)	1.6 ± 2.4	1.5–1.8	1.4 ± 2.1	1.2–1.6	1.9 ± 2.8	1.6–2.2
	Ambulatory (min)	1.8 ± 1.8	1.7–2.0	1.8 ± 1.7	1.6–2.0	1.9 ± 1.8	1.7–2.1
	Ambulatory (%)	4.1 ± 3.9	3.8–4.4	4.0 ± 3.8	3.6–4.4	4.2 ± 4.0	3.8–4.6
	Total (min)	2.6 ± 2.4	2.4–2.8	2.4 ± 2.3	2.2–2.7	2.7 ± 2.6	2.5–3.0
	Total (%)	5.7 ± 5.3	5.3–6.1	5.4 ± 5.0	4.9–5.9	6.1 ± 5.7	5.5–6.7
MVPA	Non-ambulatory (min)	3.2 ± 2.4	3.1–3.4	3.2 ± 2.4	2.9–3.4	3.3 ± 2.5	3.0–3.6
	Non-ambulatory (%)	7.2 ± 5.4	6.8–7.6	7.1 ± 5.3	6.5–7.6	7.4 ± 5.6	6.7–8.0
	Ambulatory (min)	9.0 ± 5.5	8.6–9.4	8.7 ± 5.2	8.2–9.3	9.4 ± 5.9	8.7–10.0
	Ambulatory (%)	20.1 ± 12.2	19.2–21.0	19.4 ± 11.5	18.3–20.6	20.8 ± 13.0	19.4–22.3
	Total (min)	12.3 ± 6.1	11.8–12.7	11.9 ± 5.7	11.3–12.5	12.7 ± 6.4	12.0–13.4
	Total (%)	27.3 ± 13.4	26.3–28.3	26.5 ± 12.7	25.2–27.8	28.2 ± 14.3	26.6–29.7
Sedentary time (min)		10.9 ± 7.8	10.4–11.5	11.2 ± 7.9	10.4–12.0	10.6 ± 7.6	9.7–11.4
Sedentary time (%)		24.3 ± 17.3	23.0–25.6	25.0 ± 17.5	23.2–26.7	23.5 ± 17.0	21.6–25.3
		Lower grade: 1st and 2st grade (n: 145)		Middle grade: 3rd and 4nd grade (n: 178)		Higher grade: 5th and 6th grade (n: 79)	
		Mean ± SD	95% CI	Mean ± SD	95% CI	Mean ± SD	95% CI
Age (years)		7.6 ± 0.6	7.5–7.7	9.5 ± 0.6	9.4–9.6	11.4 ± 0.5	11.3–11.5
Height (cm)		122.5 ± 6.2	121.5–123.5	133.5 ± 6.3	132.5–134.4	143.5 ± 6.8	141.9–145.0
Weight (kg)		24.0 ± 4.9	23.2–24.8	29.2 ± 5.6	28.3–30.0	35.2 ± 6.1	33.8–36.5
Body Mass Index (kg/m^2^)		15.8 ± 2.0	15.5–16.2	16.3 ± 2.2	15.9–16.6	17.0 ± 2.1	16.5–17.5
Relative body weight (%)		−0.8 ± 11.9	−2.8 - 1.1	−3.7 ± 12.1	−5.4 - -1.9	−4.5 ± 11.1	−7.0 - -2.0
Weight status (overweight and obese:%)		7 (4.8%)		10 (5.6%)		2 (2.5%)	
Weight status (thin:%)		1 (0.7%)		8(4.5%)		3 (3.8%)	
PE (min/lesson)		(PE lessons: 250)		(PE lessons: 324)		(PE lessons: 140)	
LPA	Non-ambulatory (min)	11.9 ± 5.1	11.2–12.5	11.0 ± 4.9	10.4–11.5	11.0 ± 4.3	10.3–11.7
	Non-ambulatory (%)	26.4 ± 11.4	25.0–27.8	24.4 ± 11.0	23.2–25.6	24.5 ± 9.5	22.9–26.1
	Ambulatory (min)	10.5 ± 3.9	10.0–11.0	10.0 ± 4.7	9.5–10.5	11.6 ± 5.0	10.7–12.4
	Ambulatory (%)	23.4 ± 8.6	22.3–24.5	22.2 ± 10.5	21.1–23.4	25.7 ± 11.2	23.8–27.6
	Total (min)	22.4 ± 4.7	21.8–23.0	21.0 ± 6.1	20.3–21.7	22.6 ± 4.2	21.9–23.3
	Total (%)	49.8 ± 10.4	48.5–51.1	46.6 ± 13.5	45.2–48.1	50.2 ± 9.4	48.6–51.7
MPA	Non-ambulatory (min)	2.8 ± 2.2	2.5–3.0	2.5 ± 2.1	2.3–2.8	2.0 ± 1.5	1.7–2.2
	Non-ambulatory (%)	6.1 ± 4.8	5.5–6.7	5.6 ± 4.7	5.1–6.2	4.4 ± 3.3	3.8–4.9
	Ambulatory (min)	7.9 ± 4.7	7.4–8.5	6.7 ± 4.5	6.2–7.2	7.0 ± 4.4	6.2–7.7
	Ambulatory (%)	17.7 ± 10.4	16.4–18.9	14.9 ± 10.0	13.8–16.0	15.5 ± 9.7	13.9–17.1
	Total (min)	10.7 ± 5.0	10.1–11.3	9.2 ± 5.1	8.7–9.8	8.9 ± 4.6	8.2–9.7
	Total (%)	23.8 ± 11.0	22.4–25.1	20.5 ± 11.3	19.3–21.8	19.9 ± 10.2	18.2–21.6
VPA	Non-ambulatory (min)	0.7 ± 0.9	0.6–0.9	0.8 ± 1.1	0.6–0.9	0.7 ± 1.4	0.4–0.9
	Non-ambulatory (%)	1.6 ± 2.1	1.4–1.9	1.7 ± 2.4	1.4–2.0	1.5 ± 3.0	1.0–2.0
	Ambulatory (min)	2.0 ± 1.7	1.7–2.2	1.8 ± 1.8	1.6–2.0	1.8 ± 1.9	1.5–2.1
	Ambulatory (%)	4.3 ± 3.7	3.9–4.8	4.0 ± 3.9	3.5–4.4	3.9 ± 4.1	3.3–4.6
	Total (min)	2.7 ± 2.1	2.4–3.0	2.5 ± 2.4	2.3–2.8	2.4 ± 2.8	2.0–2.9
	Total (%)	6.0 ± 4.7	5.4–6.6	5.7 ± 5.4	5.1–6.2	5.4 ± 6.3	4.4–6.5
MVPA	Non-ambulatory (min)	3.5 ± 2.5	3.2–3.8	3.3 ± 2.5	3.0–3.6	2.6 ± 2.0	2.3–3.0
	Non-ambulatory (%)	7.7 ± 5.6	7.1–8.4	7.3 ± 5.6	6.7–8.0	5.9 ± 4.4	5.1–6.6
	Ambulatory (min)	9.9 ± 5.8	9.2–10.6	8.5 ± 5.4	7.9–9.1	8.7 ± 5.1	7.9–9.6
	Ambulatory (%)	22.0 ± 12.9	20.4–23.6	18.8 ± 11.9	17.5–20.1	19.4 ± 11.4	17.5–21.3
	Total (min)	13.4 ± 6.1	12.6–14.1	11.8 ± 6.0	11.1–12.4	11.4 ± 5.8	10.4–12.4
	Total (%)	29.7 ± 13.6	28.1–31.4	26.2 ± 13.4	24.7–27.7	25.3 ± 12.8	23.2–27.5
Sedentary time (min)		9.2 ± 6.1	8.4–10.0	12.2 ± 9.1	11.2–13.2	11.0 ± 6.7	9.9–12.1
Sedentary time (%)		20.4 ± 13.5	18.8–22.1	27.2 ± 20.1	25.0–29.4	24.5 ± 14.8	22.0–27.0

Abbreviations: *CI* confidence interval, *LPA* light physical activity, *MPA* moderate physical activity, *MVPA* moderate-to-vigorous physical activity, *SD* standard deviation, *VPA* vigorous physical activity

Compared with the girls, the boys spent significantly more time in total MVPA and non-ambulatory VPA, and ambulatory LPA and MPA, and less time in non-ambulatory LPA (Table 2). However, those mean differences were approximately 1 min.

**Table 2:** Physical activity or sedentary behavior during physical education class by gender

Dependent variables	Gender	Estimated mean ± SE	Gender	Estimated mean ± SE	Gender	Estimated mean ± SE
	LPA		MPA		VPA	
Non-ambulatory (min)	Boys	10.8 ± 0.3^*^	Boys	2.4 ± 0.1	Boys	0.8 ± 0.1^*^
	Girls	12.3 ± 0.3	Girls	2.4 ± 0.1	Girls	0.6 ± 0.1
Ambulatory (min)	Boys	10.8 ± 0.3^*^	Boys	7.1 ± 0.3^*^	Boys	1.7 ± 0.1
	Girls	9.9 ± 0.3	Girls	6.4 ± 0.2	Girls	1.7 ± 0.1
Total (min)	Boys	21.6 ± 0.3	Boys	9.4 ± 0.3	Boys	2.5 ± 0.1
	Girls	22.2 ± 0.3	Girls	8.8 ± 0.3	Girls	2.2 ± 0.1
	MVPA		Sedentary time			
Non-ambulatory (min)	Boys	3.1 ± 0.1	Boys	11.5 ± 0.5		
	Girls	3.0 ± 0.1	Girls	11.8 ± 0.4		
Ambulatory (min)	Boys	8.8 ± 0.3				
	Girls	8.1 ± 0.3				
Total (min)	Boys	11.9 ± 0.4^*^				
	Girls	11.0 ± 0.3				

Adjusted for grade, relative body weight, school

Abbreviations: *LPA* light physical activity, *MPA* moderate physical activity, *MVPA* moderate-to-vigorous physical activity, *SE* standard error, *VPA* vigorous physical activity

^*^boys vs girls (*P* < 0.05)

Times in total MPA and MVPA were significantly higher and that in sedentary time was lower in the lower grade than those of other grades (Table 3). Times in ambulatory and total LPA were significantly lower for the middle grade than those for the higher grade. Times in non-ambulatory MPA and MVPA were significantly higher for the lower and middle grades than those for the higher grade. Times in ambulatory and non-ambulatory MPA were significantly higher for the lower grade than those for the middle grade.

**Table 3:** Physical activity and sedentary behavior during physical education class by grade type

Dependent variables	Grade	Estimated mean ± SE	Grade	Estimated mean ± SE	Grade	Estimated mean ± SE
	LPA		MPA		VPA	
Non-ambulatory (min)	Lower grade	12.2 ± 0.3	Lower grade	2.9 ± 0.1^a^	Lower grade	0.7 ± 0.1
	Middle grade	11.2 ± 0.3	Middle grade	2.4 ± 0.1^b^	Middle grade	0.7 ± 0.1
	Higher grade	11.2 ± 0.4	Higher grade	1.8 ± 0.2^c^	Higher grade	0.6 ± 0.1
Ambulatory (min)	Lower grade	10.3 ± 0.3	Lower grade	7.4 ± 0.3^a^	Lower grade	1.8 ± 0.1
	Middle grade	9.6 ± 0.3^a^	Middle grade	6.3 ± 0.3^b^	Middle grade	1.7 ± 0.1
	Higher grade	11.2 ± 0.4^b^	Higher grade	6.5 ± 0.4	Higher grade	1.6 ± 0.2
Total (min)	Lower grade	22.4 ± 0.4^a^	Lower grade	10.3 ± 0.3^a^	Lower grade	2.4 ± 0.2
	Middle grade	20.9 ± 0.3^b^	Middle grade	8.7 ± 0.3^b^	Middle grade	2.4 ± 0.1
	Higher grade	22.4 ± 0.5^a^	Higher grade	8.3 ± 0.4^b^	Higher grade	2.2 ± 0.2
	MVPA		Sedentary time			
Non-ambulatory (min)	Lower grade	3.5 ± 0.2^a^	Lower grade	9.8 ± 0.5^a^		
	Middle grade	3.1 ± 0.1^a^	Middle grade	13.0 ± 0.5^b^		
	Higher grade	2.5 ± 0.2^b^	Higher grade	12.1 ± 0.7^b^		
Ambulatory (min)	Lower grade	9.2 ± 0.3^a^				
	Middle grade	7.9 ± 0.3^b^				
	Higher grade	8.1 ± 0.5				
Total (min)	Lower grade	12.7 ± 0.4^a^				
	Middle grade	11.1 ± 0.3^b^				
	Higher grade	10.5 ± 0.5^b^				

Adjusted for gender, relative body weight, school

Abbreviations: *LPA* light physical activity, *MPA* moderate physical activity, *MVPA* moderate-to-vigorous physical activity, *SE* standard error, *VPA* vigorous physical activity

Different letters indicate statistically significant differences among the three grades (*P* < 0.05)

Seventy-four lessons could not be classified into any of the types of PE on the log sheets used by the class teachers, and were excluded accordingly. PE lessons that consisted of exercises for physical fitness were not conducted in the 4th and 5th grades, and expressive activities (e.g. expression, including dancing with improvisation and making a simple sequence by exploring the image, rhythm dance, folk dance) were not conducted at all in the present study. Thus, the present study compared three types of lessons (gymnastics, track and field, and ball games) (Table 4). Times in total MPA and MVPA were significantly shorter for gymnastics and for track and field than those for ball games. Time in total VPA was significantly shorter in gymnastics than those in others. Time in sedentary time was significantly longer in gymnastics than those in others.

**Table 4:** Physical activity or sedentary behavior during physical education lesson by type of lesson

Dependent variables	Type of lesson	Estimated mean ± SE	Type of lesson	Estimated mean ± SE	Type of lesson	Estimated mean ± SE
	LPA		MPA		VPA	
Non-ambulatory (min)	Gymnastics	11.7 ± 0.4^a^	Gymnastics	3.8 ± 0.2^a^	Gymnastics	0.5 ± 0.1^a^
	Track and field	12.8 ± 0.3^a^	Track and field	2.1 ± 0.1^b^	Track and field	1.2 ± 0.1^b^
	Ball games	10.4 ± 0.3^b^	Ball games	1.8 ± 0.1^b^	Ball games	0.4 ± 0.1^a^
Ambulatory (min)	Gymnastics	8.1 ± 0.5^a^	Gymnastics	4.9 ± 0.4^a^	Gymnastics	0.6 ± 0.2^a^
	Track and field	9.8 ± 0.3^b^	Track and field	5.3 ± 0.3^a^	Track and field	2.1 ± 0.1^b^
	Ball games	12.3 ± 0.3^c^	Ball games	9.3 ± 0.3^b^	Ball games	1.9 ± 0.1^b^
Total (min)	Gymnastics	19.8 ± 0.5^a^	Gymnastics	8.7 ± 0.4^a^	Gymnastics	1.1 ± 0.2^a^
	Track and field	22.6 ± 0.4^b^	Track and field	7.4 ± 0.4^b^	Track and field	3.3 ± 0.2^b^
	Ball games	22.6 ± 0.4^b^	Ball games	11.1 ± 0.3^c^	Ball games	2.3 ± 0.2^c^
	MVPA		Sedentary time			
Non-ambulatory (min)	Gymnastics	4.4 ± 0.2^a^	Gymnastics	15.4 ± 0.7^a^		
	Track and field	3.3 ± 0.2^b^	Track and field	11.7 ± 0.6^b^		
	Ball games	2.2 ± 0.2^c^	Ball games	9.0 ± 0.5^c^		
Ambulatory (min)	Gymnastics	5.5 ± 0.5^a^				
	Track and field	7.4 ± 0.4^b^				
	Ball games	11.2 ± 0.4^c^				
Total (min)	Gymnastics	9.9 ± 0.5^a^				
	Track and field	10.7 ± 0.4^a^				
	Ball games	13.4 ± 0.4^b^				

Adjusted for gender, grade, relative body weight, school

Abbreviations: *LPA* light physical activity, *MPA* moderate physical activity, *MVPA* moderate-to-vigorous physical activity, *SE* standard error, *VPA* vigorous physical activity

Different letters indicate statistically significant differences among the three types of lessons (*p* < 0.05)

The mean values of class size in each group were as follows: the smallest group, 27 ± 2 students/class; the middle group, 32 ± 1 students/class; and the largest group, 37 ± 2 students/class, respectively (from 20 to 40 students/class). Times in total MPA, total MVPA (the estimated mean values of total MVPA for the smallest group: 9.6 ± 0.5 min), ambulatory MPA and ambulatory MVPA were significantly shorter for the group with the smallest number of students than that for other groups (total MVPA for the middle group: 11.5 ± 0.4 min, the most group: 12.7 ± 0.5 min; *P* < 0.05). The time in ambulatory LPA was significantly longer for the middle and largest groups than for the smallest group. The time in sedentary time was significantly shorter for the largest group (9.6 ± 0.6 min) than for other groups (the smallest group: 14.0 ± 0.7 min, the middle group: 12.0 ± 0.5 min; *P* < 0.05). Similar results were shown when the type of PE lesson was taken into account (data not shown).

## Discussion

This study examined PAs and sedentary time that were classified as either ambulatory or non-ambulatory by using triaxial accelerometry during PE lessons and whether there were gender or grade differences among primary school children. Moreover, we determined which types of PE lessons best promote PA and reduce sedentary time. We found that times in MVPA and sedentary time during PE lessons were 27.3 ± 13.4% and 24.3 ± 17.3%, respectively. There were no obviously significant gender differences in PA and sedentary time. On the other hand, PA in the lower grade (1st and 2nd) was longer than those in other grades, and sedentary time in the lower grade was shorter than those in other grades. There were significant differences in PA and sedentary time between PE lesson types and between class sizes. Hollis et al. reported in a recent systematic review that elementary school children (aged 4–12 years) spent 32.6% (CI: 5.9–59.3%) of their PE lesson time in MVPA, as measured using accelerometers, in four studies [2]. These previous studies were from outside Japan. By comparison, the present study’s finding (27.3%) was not as high. Hollis et al. also found only one study that reported results by sex [2]. Nettlefold et al. reported that the amount and percentage of MVPA, LPA and sedentary time as evaluated with the ActiGraph GT1M was similar between girls (13.0, 14.0 and 73.0%, respectively) and boys (11.4, 14.1 and 74.5%, respectively) during PE lessons that ranged in length from 30 to 45 min per lesson, in 8–11-year-old Canadian boys and girls [6]. Moreover, Meyer et al. [16] also compared the results of primary school girls and boys, in Switzerland (9.3 ± 2.1 years). They evaluated MVPA by using the MTI/CSA 7164 and the ActiGraph GT1M in PE lessons that ranged from 45 to 50 min. They found that the children spent 32.8 ± 15.1% of their PE time in MVPA. Gender was significantly associated with MVPA: the boys spent 3.3 min (6.6%) [95% confidence interval 2.5–4.2 min (5.0–8.2%); *P* < 0.001] more in MVPA compared with the girls. Although the results of the present study also found significant differences in MVPA among grades, the mean difference was only 1 min. The difference between boys and girls was smaller in the present study compared to previous works. The percentage of MVPA in PE lessons as reported by Meyer et al. [16] was higher than that of Nettlefold et al. [6] and the present study. One of the reasons might be the difference between boys and girls in PA levels among the present study and previous studies. MEXT sets the educational curriculum guidelines for all elementary Japanese school children including the content of PE lessons, except for special classes for handicapped children [3]. PE lessons were conducted according to the guidelines under participants’ class teachers’ guidance in the present study. Thus, in the present study, gender differences among PAs in PE classes were found to be minimal. One result of this finding is that PE classes might encourage girls, who naturally tend to be inactive, to engage in PA [16–20]. In the present study, PE classes were taught by class teachers who teach a range of courses. PE classes in the majority of Japanese primary schools aren’t taught by specialized course teachers who have studied PE (only 5% in primary schools) [21]. In future studies, time in PAs and sedentary time during PE classes by specialists need to be examined.

In terms of grade, Meyer et al. [16] reported that grade (1st grade vs. 5th grade) was not associated with differences in MVPA. In the present study, MPA and MVPA in the lower grade (1st and 2nd) were longer than those in the other grades. The lower grade spent 2–3 min more in the PAs or 2–3 min less in sedentary time than did the other grades. Meyer et al. [16] reported that normal PE activities in this age range include, among other activities, playing games, coordination skills, and track and field activities. On the other hand, PE guidelines in Japan as set by MEXT clearly differentiate contents of PE lessons by grade. That is, in the lower grade, PE lessons consist mainly of active play, while in the higher grades PE lessons consist of mainly exercise or organized sports. The contents of physical fitness, however, are the same across all grades. These differences in the contents of PE lessons among the different grades in Japan might be one of the reasons for differences in MVPA among Japanese children.

Very few studies have compared MVPA between the types of exercise during PE lessons using accelerometry in primary school children [2]. In the present study, there were significant differences in PAs and sedentary time between PE lesson types. Wood and Hall [4] compared MVPA with team games (e.g., football) and movement activities (e.g., dance) as evaluated by ActiGraph GT3X or GT1M in 20 UK children aged 8–9 years. There was a significant difference between the percentage of time spent in MVPA during team games (11.4%) and movement activity (7.5%) during PE lessons. The results of the present study concur with these findings. Moreover, among the types of PA, the mean difference was nearly 1–2 min in each PA between gymnastics and ball game lessons (Table 4). However, for time in ambulatory MVPA, the mean differences were nearly 6 min (13%). Meanwhile, ambulatory PAs in track and field were significantly shorter (nearly 3–4 min [8–9%]) than those in ball game lessons. Although running is the main activity in track and field as is shown in the PE guidelines of MEXT, the present study found that ambulatory activities in track and field lessons were shorter than those of ball game lessons. The present study also found that there were also significant differences in sedentary time between the types of PE lessons. As far as we know, previous studies have never reported sedentary time as measured by an accelerometer during PE lessons, except for Nettlefold et al. [6]. Time in sedentary time during gymnastic lessons (nearly 32%) was significantly longer than those in track and field (approximately 5 min: 10%) and ball game lessons (approximately 7 min: 15%). There were also significant differences among track and field and ball game lessons. In the present study, gymnastic activities included using a steel bar and mat exercises, among other activities, while track and field activities included running long jumps, broad jumps, hurdles, high jumps, longer distance running, sprints, and relays. Ball game lessons included dodge ball, handball, basketball, volleyball, and soccer. Thus, the gymnastic and track and field lessons appeared to involve a longer time in developing skills and watching and listening to teachers, and waiting in line. In particular, the gymnastic lessons used equipment more in every lesson than did other lessons. However, the purpose of PE classes is not only to promote active PA or to improve physical fitness. Creating a positive attitude toward exercise and physical activity along with learning general motor skills about exercise are also shown to be aims of PE in the elementary school course of study [3]. In addition, students also benefit by learning cooperation, fairness, and awareness of health and safety, and by gaining a positive outlook. Thus, the guidance of teachers and discussion among students are also an important part of PE. However, according to the MEXT survey, 10.5% of 5th grade boys and 24.2% of girls were engaged in exercise and active play for less than 60 min/week outside of PE lessons [22]. The great majority (90%) of children and adolescents who exercised for less than 60 min/week did not participate in organized sports activities outside of school. The previous study showed that higher daily sedentary time may be compensated mainly by lower LPA, while the association between sedentary time and MVPA was moderate [23]. The present study found that during PE lessons, students spent nearly 20–30% of class time in sedentary time. As mentioned above, although the contents of PE lessons are fixed by MEXT, these lessons can be changed by each school, depending on differences in geography and weather conditions, availability of facilities (e.g., gym, swimming pool) and equipment [3]. Each school has to be careful not to depend too much on one type of exercise and to try to maintain a balance between different types of activities [3]. If school teachers want to improve PA and reduce sedentary time during PE, ball game lessons seem to be the easiest activity to add to the PE curriculum, as ball games encourage the most PA. In the present study, ball game lessons included dodge-ball, handball, basketball, volleyball, and soccer. These ball games might be useful to improve PA levels during PE lessons.

The MEXT survey of primary school students showed that both physical fitness and the percentage of total time in exercise and active play per week outside of PE in students in a small class were superior to those in students in medium and large classes [23]. We hypothesized that PAs during PE might also be higher in the small class than in other groups, and sedentary time might be lower. However, the present study showed the opposite results. In the MEXT survey, the smallest class size was less than 20 students, the middle class size was 20 to 59 students, and the biggest class size was 60 or more students [24]. In the present study, the range of class size was small (from 20 to 40 students/class). Further research to examine the influence of class size on PAs during PE classes with variety of class size is needed.

The current study has several limitations. Firstly, since our sample was only in urban areas, our findings may not apply to schools in other regions of Japan. Secondly, as our study was dependent on log sheets kept by class teachers to evaluate the types of PE lessons, we did not analyze data from insufficient or incomplete log sheets. Thirdly, we compared only three types of PE lessons (gymnastics, track and field, and ball games). At the same time, the strengths of our study include the use of objective measures of sedentary time, classifying ambulatory and non-ambulatory PA, and the use of a sample population of Japanese primary school children from 14 different schools.

## Conclusions

This study examined primary school students’ PA levels and sedentary time during PE by objective measurement and whether there were gender or grade differences. Moreover, we determined which PE lesson types or class sizes best support PA and reduce sedentary time. Time in MVPA and sedentary time were 27.3 and 24.3%, respectively. Time in most of the different categories of PAs in boys were significantly higher than those of girls, but predicted mean differences were only approximately 1 min. Time in PAs and sedentary time in the lower grade (1st and 2nd) were significantly higher and lower respectively than those of other grades. Moreover, the children in the current study were significantly less active during gymnastics and track and field lessons compared to ball game lessons. On the other hand, time in sedentary time during gymnastic lessons was significantly longer than those with other lessons. Children did not engage in much MVPA and also spent sedentary time during PE, but there were no gender differences. They were most active during ball game lessons, and least active in the smallest class size. Therefore, improving MVPA and reducing sedentary time during PE are important in both genders, in third grade and higher primary school children, and in PE lessons with small class sizes. PE interventions should target ball game lessons.

## Abbreviations

ANCOVA: Analysis of covariance
CI: Confidence interval
LPA: Light PA
METs: Metabolic equivalents
MEXT: The Ministry of Education, Culture, Sports, Science and Technology
MVPA: Moderate-to-vigorous physical activity
PA: Physical activity
PE: Physical education
VPA: Vigorous PA
